# Angiotensin-converting enzymes modulate aphid–plant interactions

**DOI:** 10.1038/srep08885

**Published:** 2015-03-06

**Authors:** Wei Wang, Lan Luo, Hong Lu, Shaoliang Chen, Le Kang, Feng Cui

**Affiliations:** 1 State Key Laboratory of Integrated Management of Pest Insects and Rodents, Institute of Zoology, Chinese Academy of Sciences, Beijing 100101, China; 2 College of Biological Sciences and Technology, Beijing Forestry University, Beijing 100083, China

## Abstract

Angiotensin-converting enzymes (ACEs) are key components of the renin–angiotensin system in mammals. However, the function of ACE homologs in insect saliva is unclear. Aphids presumably deliver effector proteins via saliva into plant cells to maintain a compatible insect–plant interaction. In this study, we showed that ACE modulates aphid–plant interactions by affecting feeding behavior and survival of aphids on host plants. Three ACE genes were identified from the pea aphid *Acyrthosiphon pisum* genome. ACE1 and ACE2 were highly expressed in the salivary glands and are predicted to function as secretory proteins. The ACE2 transcript level decreased in aphids fed on artificial diet compared with aphids fed on *Vicia faba*. The knockdown of the expression of each ACE by RNAi failed to affect aphid survival. When ACE1 and ACE2 were simultaneously knocked down, aphid feeding was enhanced. Aphids required less time to find the phloem sap and showed longer passive ingestion. However, the simultaneous knockdown of ACE1 and ACE2 resulted in a higher mortality rate than the control group when aphids were fed on plants. These results indicated that ACE1 and ACE2 function together to modulate *A. pisum* feeding and survival on plants.

Angiotensin-converting enzyme (dipeptidyl carboxypeptidase, ACE, EC 3.4.15.1) is a zinc-metallopeptidase found on the surfaces of cells from various mammalian tissues^1^. The interaction of ACE with zinc is directly controlled by the active site HEXXH^2^. This enzyme removes dipeptides from the C-terminus of short oligopeptides^3^. ACE is well studied in mammals, and regulates the blood pressure and electrolyte homeostasis, thereby serving as a key component of the renin–angiotensin system. Two distinct ACE forms exist in mammals, as follows: somatic ACE, which contains two highly similar domains (N- and C-domains); and testicular ACE, which is restricted to spermatid and spermatozoon development and possesses a single domain that is identical to the C-domain of somatic ACE^1^. Insect ACEs have similar enzymatic properties to mammalian ACE, but insect ACEs structurally differ because of their soluble and poorly glycosylated proteins^4^,^5^,^6^.

The functions and substrate specificity of insect ACE-homologs have not been adequately investigated, even if ample evidence on the importance of ACEs in normal growth and development has been found in several insect species^5^,^7^,^8^,^9^. Insects have an open circulatory system, and insect ACEs presumably do not serve as components of a renin–angiotensin system as the ACEs found in vertebrates. Insect ACEs identified to date resemble the testicular form of vertebrate ACEs because they possess a single domain^10^. ACEs are widely distributed in different insect tissues and cell types. Therefore, the biological functions of ACEs may be diversified. In several insect species, ACE is enriched in the testes and affects male fertility^11^. The abundance of ACE in gut tissues of *Spodoptera littoralis*, *Lucilia cuprina*, and *Haematobia irritans exigua* suggests that insect ACE has a function in gut hormone processing^10^,^12^. In insect brain tissues, ACE is localized in the neuropile regions and neurosecretory cells, both of which probably function in neuropeptide processing^10^,^13^. Immune challenge resulted in a 10-fold increase of the ACE transcripts in the hemocytes of locust *Locusta migratoria*, thereby indicating that ACE functions in cellular defense^14^.

Aphids constitute a large group of piercing–sucking insects that feed on sieve elements^15^. Knowledge on the nature of proteins in aphid saliva and salivary glands has increased in recent years. Five enzymes (glucose oxidase, glucose dehydrogenase, NADH dehydrogenase, α-glucosidase, and α-amylase) have been detected in the saliva of green peach aphid *Myzus persicae*^16^. Nine proteins have been identified in the saliva of the pea aphid *Acyrthosiphon pisum*, four of which were ACE, M1 zinc-dependent metalloprotease, glucose-methanol-choline-oxidoreductase, and regucalcin^17^. A catalog of candidate effector proteins from the *A. pisum* salivary glands has been generated, and 42 transcripts were enriched in the salivary glands, including ACE^18^. Effector proteins are defined as all pathogen/pest proteins that alter the host-cell structure and function. These alterations could facilitate infections, trigger defense responses, or both^19^. However, limited information is available on the functions of effector proteins in aphid–plant interactions. The aphid-specific protein c002 enhances aphid feeding and colonization^20^,^21^,^22^ in a plant species-specific manner^23^. Aphids spend little time in contact with the phloem sap in sieve elements with the knockdown of *c002* transcripts^22^. An unknown *A. pisum* salivary protein (ACYPI39568) belonging to an aphid-specific cysteine-rich protein family has been found as a zinc-binding protein. Aphids require more ACYPI39568 when feeding on plants than when feeding on artificial diets. However, ACYPI39568 does not affect the survival rate of aphids fed on plants^24^. The *M. persicae* effector Mp10 induces chlorosis and local cell death in plants^20^. Calcium-binding proteins prevent phloem sieve cell plugging upon mechanical damage caused by aphid stylets^25^.

Aside from the *A. pisum* salivary glands, ACE transcripts have also been detected in whitefly (*Bemisia tabaci* complex) primary salivary glands^26^. However, the role of ACE as an effector in modulating insect–plant interactions has not been previously demonstrated. Based on the enriched ACE expression in aphid salivary glands and the presence of ACE in aphid saliva, ACEs presumably enter the plant phloem and possibly other tissues during feeding. In this study, we aimed to determine whether ACE putatively functions as an effector protein to modulate aphid–plant interactions.

## Results

### Characteristics of ACE genes and proteins

When the sequence of ACE highly expressed in aphid salivary glands^18^, which is referred in the present study as ACE1 (XM_001951605 in NCBI and ACYPI000733 in aphidbase), was used to perform BLAST against the *A. pisum* genome, two other ACE homolog genes, namely, ACE2 (XM_001943123 in NCBI and ACYPI007204 in aphidbase) and ACE3 (XM_001949361 in NCBI and ACYPI005682 in aphidbase) were detected. The three ACE genes are located in different scaffolds of the aphid genome, particularly scaffolds EQ116294 (ACE1), EQ116276 (ACE2), and EQ113364 (ACE3). ACE1 and ACE3 are encoded by a complementary sense DNA, and ACE2 is encoded by sense DNA. ACE3 is incomplete at the 3' end of the open reading frame (ORF). The ORFs of the three ACE genes were cloned, and their lengths were 1914, 1884, and 1785 base pair (bp). A longer ACE3 transcript (i.e., 1865 bp) was found in one of the six sequenced clones. When the ORFs were compared with the *A. pisum* genome, the ACE gene structures were determined. Eleven exons in ACE1 and ACE2 were noted, and at least 13 exons in ACE3 were observed (Figure S1). The intron patterns, including position and length, differed among the three ACE genes. The ACE1 first exon was located downstream of the last exon on the genome, thereby reflecting the aberrant genome assembly. The longer ACE3 transcript was derived from an alternative splicing of the fourth intron (80 bp); this transcript was transcribed, and joined the fourth and fifth exons into one exon (Figure S1). The transcription of the additional 80 bp led to an earlier translation stoppage.

**Figure 1:**
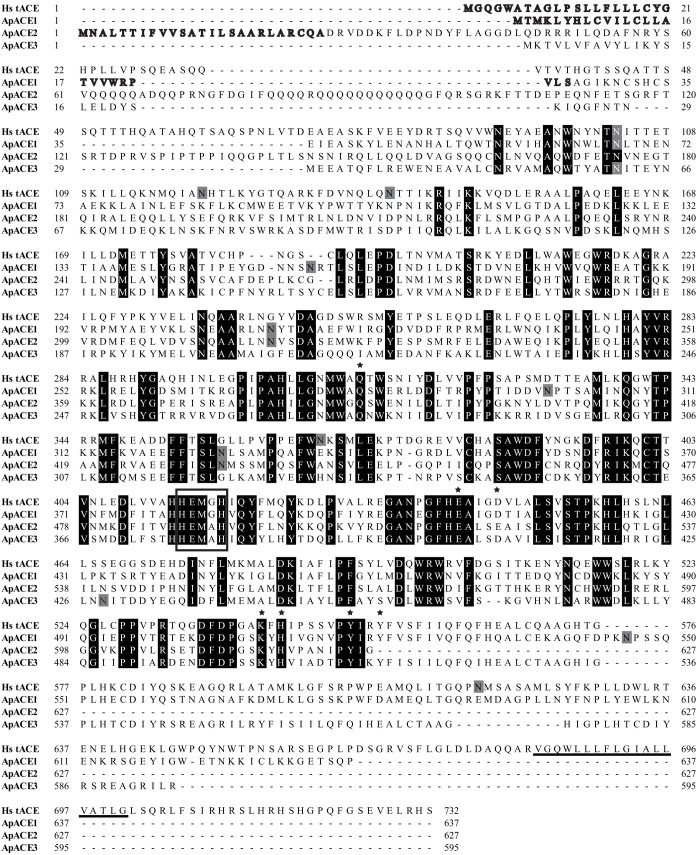
Alignment of amino acid sequences of ACEs from *A. pisum* (ApACE1- ApACE3) and human testicular ACE (Hs tACE, NP_690043). Predicted secretion of signal peptides are in bold letters. Predicted hydrophobic region of the human enzyme is underlined. Active site sequence is boxed. Amino acids with important functions in enzyme activities and substrate/inhibitor bindings are marked with stars. *N*-glycosylation sites are shaded grey. Positions of identity among all four sequences are shaded black.

A secretory signal peptide was predicted to possess putative cleavage site between residues Ser^25^ and Ala^26^ for ACE1 and between residues Ala^25^ and Asp^26^ for ACE2 (Figure 1). No other hydrophobic region was present, thereby suggesting that ACE1 and ACE2 are secreted proteins. No secretion signal was predicted for ACE3, thereby indicating that ACE3 is not a secreted protein. The predicted molecular weights of mature ACE1 and ACE2 proteins were 71.6 and 70.3 kDa, respectively. The actual molecular weights of the proteins could be higher because six and two putative *N*-glycosylation sites were predicted for ACE1 and ACE2, respectively. The possible *N*-glycosylation sites were not conserved in the three ACEs (Figure 1). The pairwise identities of the amino acid sequences of the three ACEs ranged from 30.5% to 38.6%. All these sequences contain the active site motif, HEXXH, which is a characteristic of ACEs and is required for enzyme activity (Figure 1).

From a phylogenetic standpoint, the three *A. pisum* ACEs belonged to distinct clusters (Figure S2). *A. pisum* ACE1 was most similar to *L. migratoria* ACE and was in a cluster with two *Bombyx mori* ACEs and one *S. littoralis* ACE. Most members of this cluster are soluble proteins. *A. pisum* ACE2 was most similar to *Drosophila melanogaster* ACE3, which is a membrane-bound protein. *A. pisum* ACE3 was out-grouped from the other insect homologs, thereby indicating that ACE3 has a function distinct from other ACEs.

### Temporal and spatial expressions of the three ACEs

**Figure 2:**
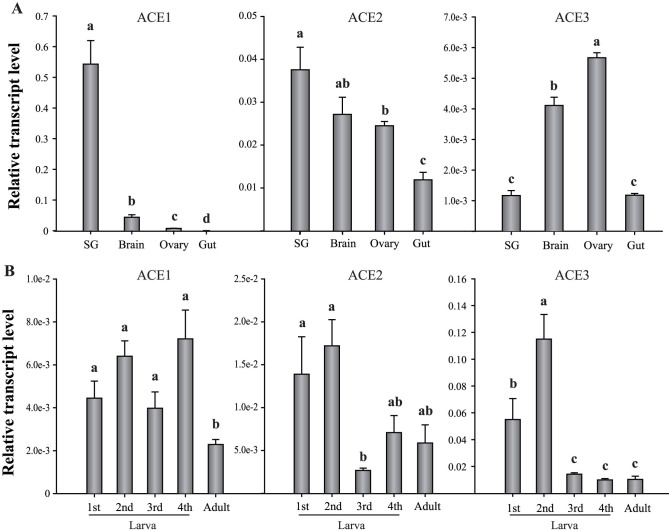
Temporal and spatial expressions of *A. pisum* ACEs measured with real-time qPCR. (A) Expressions in salivary glands (SG), brain, ovaries, and gut. (B) Expression in larval and adult stages. Values are represented as mean ± SEM. Letters above the columns indicate the comparison among groups evaluated with ANOVA using SPSS 17.0.

RNAs from the salivary glands, brains, ovaries, and whole guts of aphids were analyzed using real-time quantitative PCR (qPCR) to determine the transcript levels of the three ACEs (Figure 2A). ACE1 was mainly expressed in the salivary glands, whereas ACE2 was most highly expressed in the salivary glands, brain, and ovaries. Moreover, ACE3 was most highly expressed in the ovaries and brain. ACE1 showed higher expressions during the larval stages than during the adult stages, and ACE3 was highly expressed in the second instar larvae. Meanwhile, ACE2 was comparatively evenly expressed throughout the aphid's development (Figure 2B).

### Transcript levels of ACEs in plant-fed and diet-fed aphids

**Figure 3:**
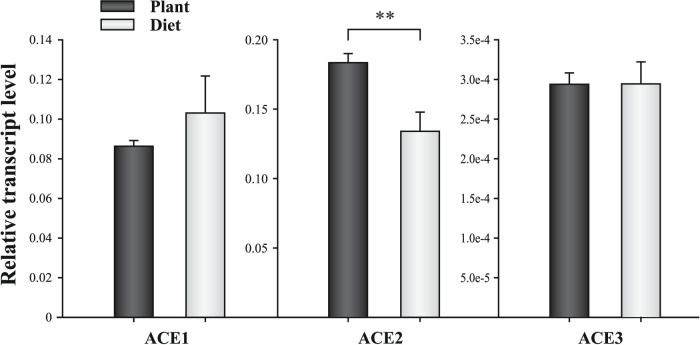
Transcript levels of *A. pisum* ACEs in the heads (containing the salivary glands) of diet- and plant-fed aphids. **, *P* < 0.01.

The transcript levels of the three ACEs in aphid heads, which contain the salivary glands, were compared between plant-fed and artificial diet-fed aphids by using qPCR. The ACE2 transcription level decreased by 27% when aphids were fed on artificial diet compared with aphids fed on *Vicia faba* (Figure 3). This result indicated that a higher amount of ACE2 was required by aphids during interactions with host plants. No significant change was observed in the ACE1 or ACE3 transcript level when the aphids were fed with the two food types (Figure 3).

### Survival rates of aphids after ACE expression interference

**Figure 4:**
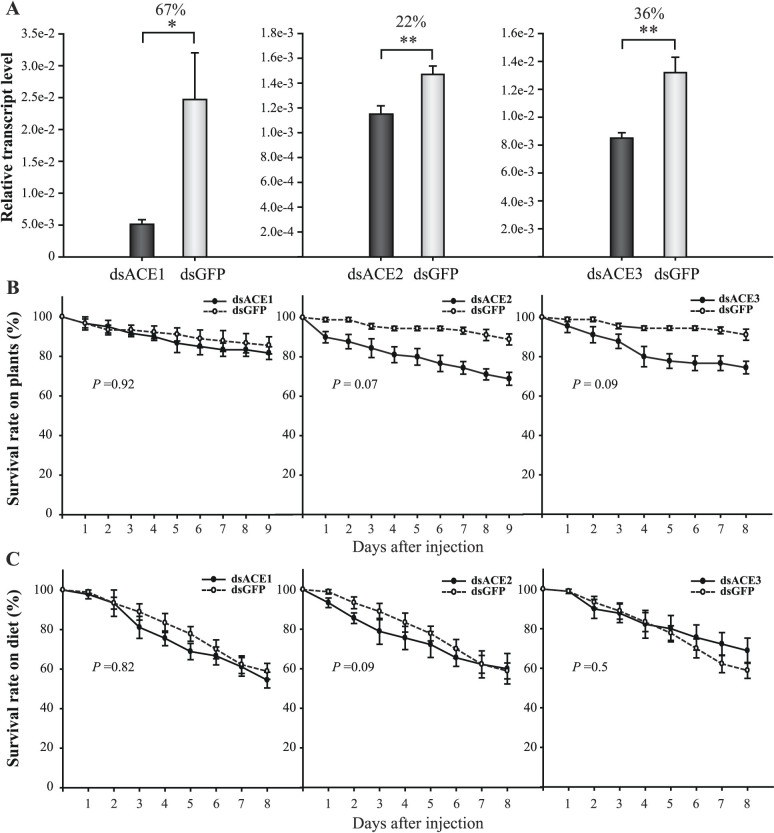
Effect of single ACE to aphid survival after dsRNA injection. (A) Transcript levels of ACEs in whole bodies of aphids after dsRNA injection. Interference ratios are indicated above the columns. *, *P* < 0.05. **, *P* < 0.01. (B and C) Survival curves of aphids feeding on *V. faba* or on artificial diet after dsRNA injection. *P* values of the difference between curves of interference and control groups are indicated.

**Figure 5:**
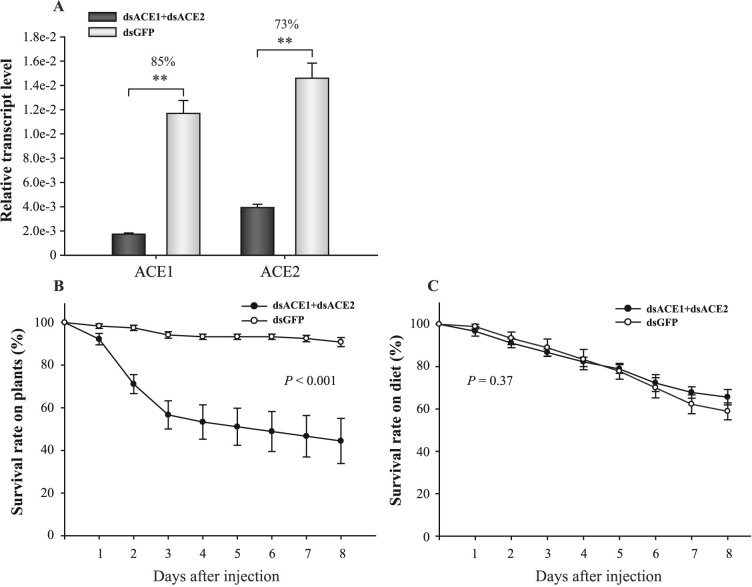
Effects of ACE1 and ACE2 on aphid survival after simultaneous knockdown. (A) ACE1 and ACE2 transcript levels in whole bodies of aphids after injection of 1:1 mixture of dsACE1-RNA and dsACE2-RNA. Interference ratios are indicated above the columns. **, *P* < 0.01. (B and C) Survival curves of aphids feeding on *V. faba* or on artificial diet after simultaneous knockdown. *P* values of the difference between curves of interference and control groups are indicated.

The dsRNA of each ACE transcript was injected in the *A. pisum* third instar. The transcript knockdown efficiencies in whole aphid bodies were 67%, 22%, and 36% for ACE1, ACE2, and ACE3, respectively (Figure 4A). No cross interference was observed when each ACE gene was knocked down (Figure S3). When ACE1, ACE2, or ACE3 was individually knocked down, no significant difference in survival was observed from that of the control (with dsGFP-RNA injection) whether the aphids were fed on *V. faba* (Figure 4B) or artificial diet (Figure 4C) (*P* > 0.05). Because ACE1 and ACE2 were highly expressed in the salivary glands, we knocked down the transcripts of the two genes simultaneously with 69 ng dsRNA for each gene. The interference levels were 85% and 73% for ACE1 and ACE2, respectively (Figure 5A), and such levels were significantly higher than that of each gene interference, especially ACE2 (Figure 4A). This double interference resulted in a significantly lower survival rate than in the control group when aphids were fed on *V. faba* (Figure 5B). However, this phenomenon was not observed when aphids were fed on artificial diet (Figure 5C).

### Feeding behavior after ACE expression interference

**Figure 6:**
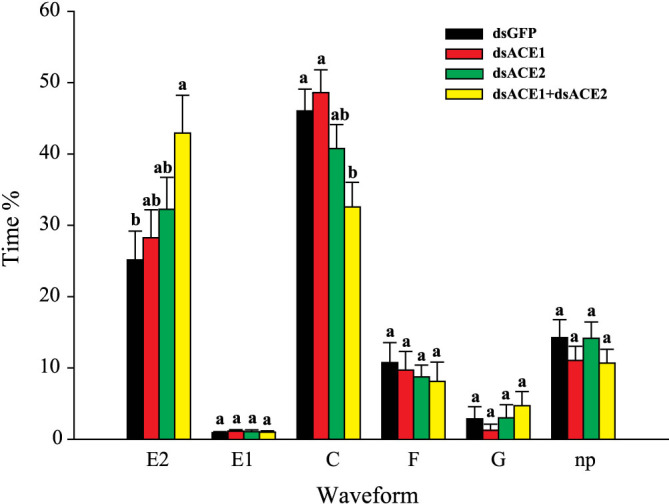
Comparison of EPG waveforms of *A. pisum* feeding behavior after dsRNA injection. E1, watery salivation. E2, passive ingestion. C, probing plant cells. F, derailed stylet. G, drinking from xylem. np, non-probing. Time ratios (time spent in each waveform divided by 8 h) are reported as mean ± SEM. Letters above columns indicate the comparison within groups of the same waveform evaluated by ANOVA using SPSS 17.0.

When either ACE1 or ACE2 transcript was knocked down, no significant change was observed in the feeding behavior of aphids on plants compared with the dsGFP-RNA-injected aphids (i.e., the control) (Figure 6). However, when ACE1 and ACE2 transcripts were simultaneously knocked down, the total duration of passive ingestion (E2) significantly increased compared with the control. However, this value did not exceed that of the dsACE1 or dsACE2 group. The time of probing individual plant cells (C) was significantly less than that of the control (Figure 6). These data indicated that the double knockdown aphids spent less time seeking phloem sap than the control aphids. No significant variation was observed in the time spent in watery salivation (E1), non-probing (np), overcoming derailed stylet mechanics (F), or drinking from xylem (G) (Figure 6).

### Reproduction ability after ACE expression interference

The fecundity within 7 d of the adult stage was compared between the ACE knockdown and control groups. The fecundity did not significantly change when any of the three ACEs was knocked down (*P* > 0.05) (Figure S4).

## Discussion

ACEs apparently have several functions in insects. These enzymes have been detected in the testis, mid-gut, brain, hemocyte, and venom of insects^10^,^11^,^12^,^13^,^14^,^27^. In aphids, ACE transcripts are enriched in salivary glands, and the protein has been detected in saliva^17^,^18^. Aphids release saliva containing host plant-modulating effectors^19^. The functions of ACEs as effectors in mediating compatible interactions between aphids and their host plants remain unknown. In this study, three putative ACEs from *A. pisum* genome were identified, and the synergistic functions of ACE1 and ACE2 in facilitating the survival of aphids on host plants were demonstrated. Aside from broadening the established knowledge regarding the function of this enzyme in mammal renin–angiotensin system and insect physiological processes, the present study also reveals new insights into the molecular basis of insect–plant interactions.

The functions of the three ACEs in *A. pisum* are apparently redundant, but these functions are diverse. Although ACE1 is excessively expressed in the salivary glands, aphids do not require additional ACE1 when fed on plants rather than on artificial diet. This fact reflects the constitutional expression mode of the gene in salivary glands. ACE2 expressions in the salivary glands, brain, and ovaries are comparable. ACE2 expression is more prevalent than that of the other two ACEs. Additional ACE2 in salivary glands and/or brain are induced upon aphid feeding on plants, thereby exhibiting an inducible expression mode. However, decreasing the expression of ACE2 alone does not affect the infestation of aphids on plants. Only the simultaneous interferences of the constitutional ACE1 and inducible ACE2 expression have lethal effects, thereby indicating that ACE1 and ACE2 simultaneously function in the salivary glands to successfully secure aphids on host plants. ACE3 is predominantly expressed in the ovaries and brain, but this gene does not affect aphid reproduction when knocked down. This enzyme probably functions in the neuropeptide processing in aphid brain tissues. Another possibility suggests that ACE3 could simultaneously function with ACE2 during aphid reproduction because of their abundant transcripts in the ovaries. ACEs affect female insect reproduction. ACE inhibitors reduce the fecundity of the female mosquito *Anopheles stephensi*^28^. The presence of yolk protein-related ACE substrates in the ovaries of *Neobellieria bullata* suggests that ACE is a regulator of the vitellogenic processes^29^.

ACE is a member of the M2 metalloprotease family, which is involved in the digestion of small peptides, such as hormones or neuropeptides. Plant phloem sap contains sugars, water, minerals, amino acids, and plant hormones^30^,^31^. Among plant hormones, a certain type of peptide hormone is released as a signal molecule to induce defense responses when plants are damaged by herbivorous insects. For example, the most known peptide hormone of Solanaceae plants, systemin, has been detected inside the sieve elements of the phloem and transported throughout the plant as a long-distance signal to activate chemical defenses against herbivores^32^,^33^. Systemin and jasmonic acid activate the expressions of proteinase inhibitors and other defense-related genes through the same signaling pathway^34^. Plant defense against aphids follows the plant–pathogen model, in which compatible hosts are manipulated by the secretion of effector molecules that suppress (or otherwise interfere with) the plant's innate immunity defense^35^. Further research is required to determine whether ACEs in aphid saliva can hydrolyze systemin or other signal molecules of peptide hormones that induce plant immune reactions.

Aside from the possible involvement in plant peptide hormones, ACEs hydrolyze diverse peptide hormones/transmitters, such as leucokinin, locustatachykinin, and allatostatin within insects^36^. Leucokinin and locustatachykinin are myotropic neuropeptides. Leucokinin increases the Malpighian tubule fluid secretion and hindgut motility and regulates insect meal size^37^,^38^. Allatostatins suppress food intake or inhibit feeding behavior in insects^39^,^40^. In the present study, the simultaneous ACE1 and ACE2 knockdowns increased the fluency of aphid feeding (longer passive ingestion and shorter probing time). This study is the first to report that a salivary gland protein exerts a negative effect on the feeding behavior of aphids. The well-known effector protein, c002^21^,^22^, and another effector protein, Armet^41^, from *A. pisum* are essential in maintaining a normal feeding behavior. The c002 or Armet knockdowns significantly reduced the contact time of aphids with the phloem sap in sieve elements. Therefore, the regulation of the aphid feeding behavior by ACEs that act on endogenous neuropeptides is highly possible.

The physiological substrates of ACEs hydrolyzed in vivo have not been fully elucidated in insects, except for the ovary-derived ACE interactive factor *Neb*-ODAIF from *N. bullata*^29^. ACEs might be involved in the processing of biologically active peptides in aphid saliva or salivary glands. To date, information on the possible ACE substrates in aphid salivary glands is lacking. Strong ACE activities and antiserum immunoreactions have been reported in the venom from the endoparasitic wasp, *Pimpla hypochondriaca*^27^. An insect homolog of the known mammalian ACE substrate, bradykinin, has been detected in the venom of several solitary scoliid wasps^42^. Further research is needed to determine whether ACE substrates exist in aphid saliva or salivary glands, and to elucidate the mechanisms undergone by the turnover product to facilitate the survival of aphids on plants.

ACE homologs from several insects have been studied for their functions, but only a single ACE gene is usually reported in each insect, except in *D. melanogaster* and *B. mori*. According to the phylogenetic analysis, *A. pisum* ACE1 is most similar to *L. migratoria* ACE (AAR85358), *B. mori* ACEs (BAA97657, BAH23569), and *S. littoralis* ACE (ABW34729) (Figure S2). *L. migratoria* ACE (AAR85358) is most highly expressed in the testes, followed by the brain, midgut, ovaries, and hemocytes; this gene is least expressed in fat bodies^14^. *B. mori* ACE (BAA97657) is abundant in wing discs at certain developmental stages, and is induced by exposure to 20-hydroxyecdysone^43^. *S. littoralis* ACE (ABW34729) is a presumably soluble enzyme, and is expressed throughout the insect life cycle, especially in the brain, gut, and fat body tissues of the last larval stage^10^. The present study showed that *A. pisum* ACE1 was most highly expressed in salivary glands, followed by the brain, ovaries, and gut. Although these ACE homologs have considerably similar sequences, their functions could have been diversified within each insect species during evolution.

Unlike mammalian ACEs, which are membrane-bound proteins, insect ACEs are generally secreted as soluble proteins^44^. Similar to other insect ACEs, *A. pisum* ACE1 and ACE2 are putative secretory proteins based on the predicted secretory signal peptide at the N-terminus and no other hydrophobic region. However, *A. pisum* ACE1 is suspected from a non-salivary gland origin and fat bodies can be its source^17^. In this study, we illustrated the predominant ACE1 transcripts in salivary glands in comparison with those in the brain, ovaries, and gut. We excluded the possibility of a non-salivary gland origin for ACE1. The extracellular secretion of *A. pisum* ACE3 was not possible because no secretory signal peptide was predicted at the N-terminus. The cellular location of this enzyme is difficult to determine because of the limited information on the C-terminus. The presence of membrane-bound ACEs in insects is questionable. A membrane-bound ACE is enriched in the salivary glands of the adult females of the cattle tick *Boophilus microplus*^45^.

## Methods

### Aphids

*A. pisum* subjects were collected from peas (*Pisum sativum*) in 2010 at Yuxi, Yunnan Province, China, and reared on fava beans (*V. faba*) in incubators at 21 ± 1°C and 60 ± 5% relative humidity. The photoperiod was 16 h light/8 h dark.

### RNA isolation and cDNA synthesis

Total RNA was extracted from the whole body of aphids using a RNeasy mini kit (Qiagen, Valencia, CA, USA) or from dissected tissues using TRIZOL (Invitrogen, Carlsbad, CA, USA) according to the manufacturers' protocols. RNA was treated with TURBO DNA-free kit (Ambion, Austin, TX, USA) to eliminate genomic DNA contamination. Afterward, RNA was reverse-transcribed to cDNA using SuperScript™ III first-strand synthesis system for RT-PCR (Invitrogen, Carlsbad, CA, USA) according to the manufacturers' instructions.

### ORF cloning of three ACE cDNA and protein sequence analysis

Full-length ORFs of ACE1, ACE2, and ACE3 were amplified from a whole body cDNA library with primer pairs, ACE1-F and ACE1-R, ACE2-F and ACE2-R, and ACE3-F and ACE3-R, respectively (Table S1), using Platinum® Taq DNA Polymerase High Fidelity (Invitrogen, Carlsbad, CA, USA). The PCR products were connected using the pGEM-T easy vector (Promega, San Luis Obispo, CA, USA) and transfected into DH5α cells for sequencing. Protein sequences were deduced from the sequenced ORFs and analyzed using SignalP (www.cbs.dtu.dk/services/SignalP) and SOSUI (http://bp.nuap.nagoya-u.ac.jp/sosui) servers that identify the signal peptide and predict membrane protein, respectively.

### Phylogenetic analysis

Homologous proteins from other species were identified using BlastP software at NCBI (http://blast.ncbi.nlm.nih.gov/Blast.cgi) and were aligned using ClustalW at EBI (http://www.ebi.ac.uk/Tools/msa/clustalw2/). A phylogenetic tree was constructed with the neighbor-joining method using a matrix of pair-wise distances estimated under Poisson model for amino acid sequences through MEGA 5 software. Bootstrap analysis (1000 replicates) was performed to evaluate the internal support of the tree topology with a 70% cut-off value.

### Quantification of ACE gene expressions in tissues and developmental stages

Four tissues (brain, salivary glands, ovaries, and digestive gut) were collected from approximately 20 adult aphids for RNA extraction. Six replicates for each tissue were prepared. RNA was also isolated from the first to fourth instars and from adult aphids. Six replicates and five individuals per replicate were prepared for each developmental stage.

qPCR was performed to quantify the transcript levels of the three ACE genes in the four tissues and in various developmental stages. Three pairs of primers, namely, ACE1-qPCR-F and ACE1-qPCR-R, ACE2-qPCR-F and ACE2-qPCR-R, and ACE3-qPCR-F and ACE3-qPCR-R, were designed to amplify 214, 286, and 217 base fragments of ACE1, ACE2, and ACE3, respectively (Table S1). Ribosomal protein L27 transcript (CN584974 in GenBank) was amplified using L27-qPCR-F and L27-qPCR-R as internal controls (Table S1). qPCR was performed on a Roche LightCycler 480 (Roche, Mannheim, Germany) with cycling conditions of 95°C for 2 min, 40 cycles of 95°C for 20 s, 55°C for 20 s, and 68°C for 20 s, followed by one cycle of 95°C for 30 s, 58°C for 30 s, and 95°C for 10 s to determine the melting curve. PCR products were sequenced to confirm the identity of the amplified genes. Differences in the transcript levels were analyzed using one-way ANOVA for multiple comparisons with SPSS 17.0 software. The results were presented as mean ± SEM.

### Comparison of ACE transcripts in diet- and plant-fed aphids

Five adult aphids were placed on *V. faba* for 24 h feeding. Another group fed on artificial diet^46^ sealed between two layers of parafilm and stretched over a sterile plastic cap (Falcon, Primaria, NJ, USA) for 24 h. The transcript levels of the three ACEs in the heads (containing the salivary glands) of diet- and plant-fed aphids were compared using qPCR. Five aphid heads were included in a repeat, and six repeats were prepared. The values were reported as mean ± SEM, and the differences were evaluated with *t*-test by using SPSS 17.0.

### Gene interference by double-stranded RNA (dsRNA) injection

PCR primers with T7 promoter sequences were used to prepare the dsRNA of the three ACE genes. Pairs of primers for specific amplification of dsACEs were designed as follows: ACE1-dsRNA-F and ACE1-dsRNA-R for a 313 base dsACE1, ACE2-dsRNA-F and ACE2-dsRNA-R for a 468 base dsACE2, and ACE3-dsRNA-F and ACE3-dsRNA-R for a 330 base dsACE3 (Table S1). A 420 bp dsRNA for green fluorescent protein (GFP) was amplified using primers GFP-dsRNA-F and GFP-dsRNA-R as negative controls (Table S1). dsRNA was generated using T7 RiboMAX Express RNAi system, and was purified using Wizard SV Gel and PCR cleanup system (Promega, Madison, Wisconsin, USA) following the manufacturer's protocols.

dsRNA injection was performed on third instar aphids. The developmental stage of injected aphids was synchronized by collecting the first instar aphids and rearing these aphids at 23°C for 3 d until the third instar stage. The larvae were immobilized on ice, and 23 nL of 6 μg/μL dsRNA was delivered into the hemolymph from the dorsal abdomens by microinjection using a glass needle and Nanoliter 2000 (World Precision Instruments, Sarasota, Florida, USA). For the simultaneous knockdowns of ACE1 and ACE2, 23 nL of a 1:1 mixture of dsACE1-RNA and dsACE2-RNA (each at 6 μg/μL) were injected. Six groups of the third instar aphids, with 15 individuals in each group, were injected and subsequently reared on *V. faba* plants or on artificial diet^46^. The mortality of aphids within several hours after injection was considered a result of mechanical damage, and dead aphids were removed from the experiment. The interference efficiency in the entire body was checked on the third day after injection of dsRNA using qPCR.

### Survival curve and reproduction analysis

Survivals of aphids that fed on plants or artificial diet were recorded every 24 h after dsRNA injection and were reported as mean ± SEM. The survival curves of knockdown and control groups on *V. faba* or artificial diet were statistically compared via the Kaplan–Meier method using Log Rank (Mantel–Cox) test in SPSS 17.0. The average numbers of offspring and 10 aphids as a group were recorded within 7 d of the adult stage. Six groups of data were collected for comparing the dsACE-injected aphids with the dsGFP-injected aphids with *t*-test using SPSS 17.0.

### Feeding behavior measurement

The electrical penetration graph (EPG) technique was performed to analyze the feeding behavior of individual aphids on *V. faba* plants according to the procedure described by Mutti et al.^22^. The feeding behavior of aphids injected with dsGFP-RNA, dsACE1-RNA, dsACE2-RNA, or mixture of dsACE1 and dsACE2 was continuously monitored for 8 h on the third day after injection. In total, 23, 30, 28, and 25 valid data sets were collected for the abovementioned groups of aphids. The EPG waveforms were recorded using a Giga amplifier series, GIGA-8 model (EPG-Systems, Wageningen, Netherlands). Six waveforms, namely, E1 (watery salivation), E2 (passive ingestion), C (probing plant cells), F (derailed stylet), G (drinking from xylem), and np (non-probing), were analyzed with the software Stylet+ of the same company, and their time ratios (the time spent in each waveform divided by 8 h) were reported as mean ± SEM. Differences among the four groups were statistically analyzed with one-way ANOVA for multiple comparisons using SPSS 17.0.

## Author Contributions

W.W. and H.L. performed the qPCR analysis and analyzed the data. L.L. conducted EPG and dsRNA injection. S.C. and L.K. helped in supervising and revising the manuscript. F.C. designed and wrote the manuscript.

## Supplementary Material

Supplementary Information Supplementary information
