# Supplementary material for: Angiotensin-converting enzymes modulate aphid–plant interactions
Source: Sci Rep. 2015 Mar 6;5:8885. doi: 10.1038/srep08885 (PMC4351530; doi:10.1038/srep08885)
Supplement: Supplementary Information [file srep08885-s1.pdf]

Angiotensin-converting enzymes modulate aphid–plant interactions

Wei Wang,[1,2#] Lan Luo,[1#] Hong Lu,[1] Shaoliang Chen,[2] Le Kang,[1] Feng Cui[1*]

[1]State Key Laboratory of Integrated Management of Pest Insects and Rodents, Institute of Zoology, Chinese Academy of Sciences, Beijing 100101, China

[2]College of Biological Sciences and Technology, Beijing Forestry University, Beijing 100083, China

[*]Correspondence author: Feng Cui, State Key Laboratory of Integrated Management of Pest Insects and Rodents, Institute of Zoology, Chinese Academy of Sciences, Beijing 100101, China. Phone: 86-10-64807218. Fax: 86-10-64807099. Email: cuif@ioz.ac.cn.

[#] These authors contributed equally to this work.

Supplementary Information

Figure S1. Gene structures of *A. pisum* ACEs on genome scaffolds. Grey arrows indicate exons (E) and transcriptional direction. An alternative splicing occurred at the fourth intron (I4) of the ACE3 gene. The fourth and fifth extrons (E4 and E5) were joined into one extron in the longer transcript.

Figure S2. Phylogenetic tree of ACE family in several insect species. The bootstrap values larger than 70% are shown at the nodes. The circle indicates an N-terminal

secretory signal peptide. The square indicates the C-terminal membrane anchor. The triangle indicates that the ACE function has been studied. Ap, *A. pisum*; Dm, *D. melanogaster*; Hs, *Homo sapiens*; Lm, *L. migratoria*; Bm, *B. mori*; Hi, *H. irritans*; and Sl, *S. littoralis*. The GenBank accession number of each sequence is in bracket.

Figure S3. Cross interference on the transcript levels of other ACE genes when each ACE gene was knocked down.

Figure S4. Fecundity of the adult stage within 7 d after injection of dsACE-RNA or dsGFP-RNA.

Table S1. Primers used in the study.

Angiotensin-converting enzymes modulate aphid – plant interactions

Wei Wang, Lan Luo, Hong Lu, Shaoliang Chen, Le Kang, Feng Cui

Figure S1

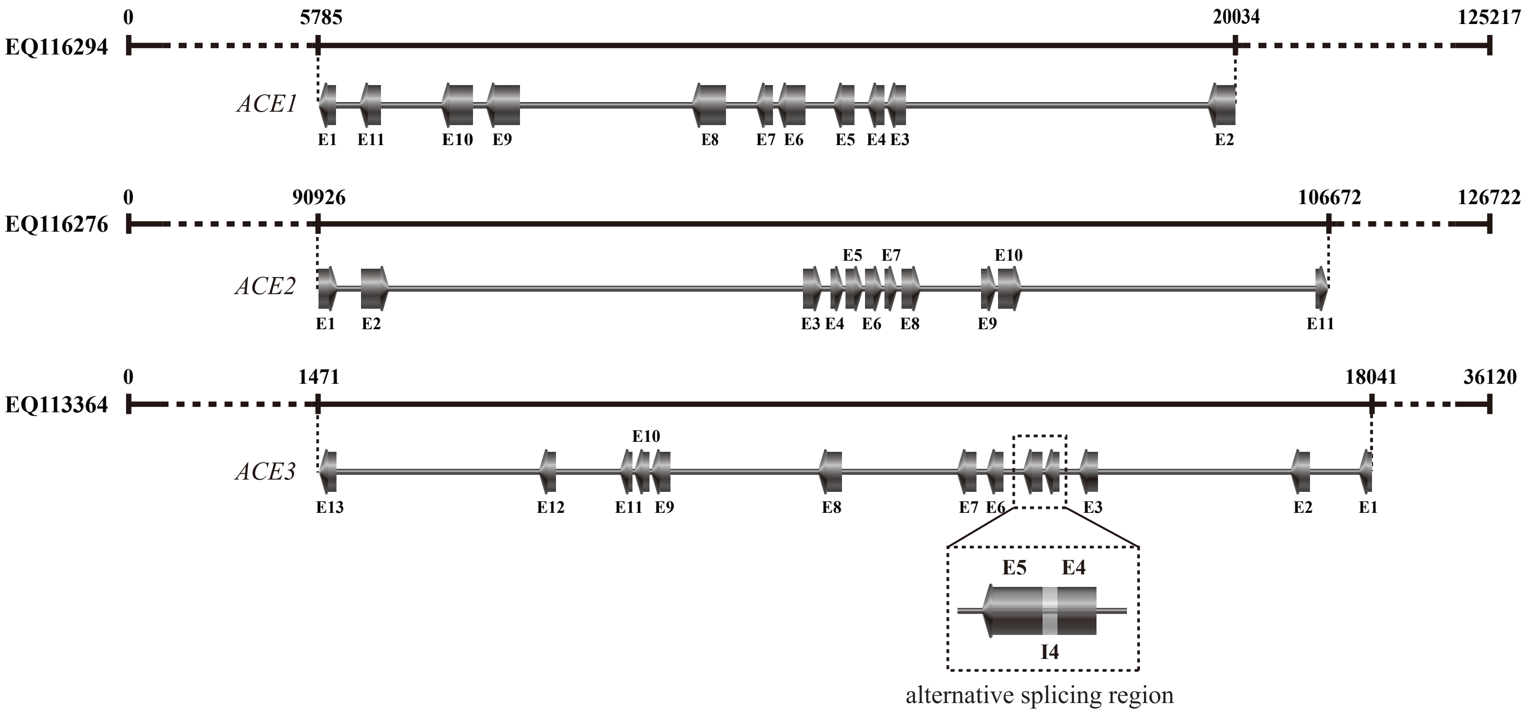

Angiotensin-converting enzymes modulate aphid – plant interactions

Wei Wang, Lan Luo, Hong Lu, Shaoliang Chen, Le Kang, Feng Cui

Figure S2

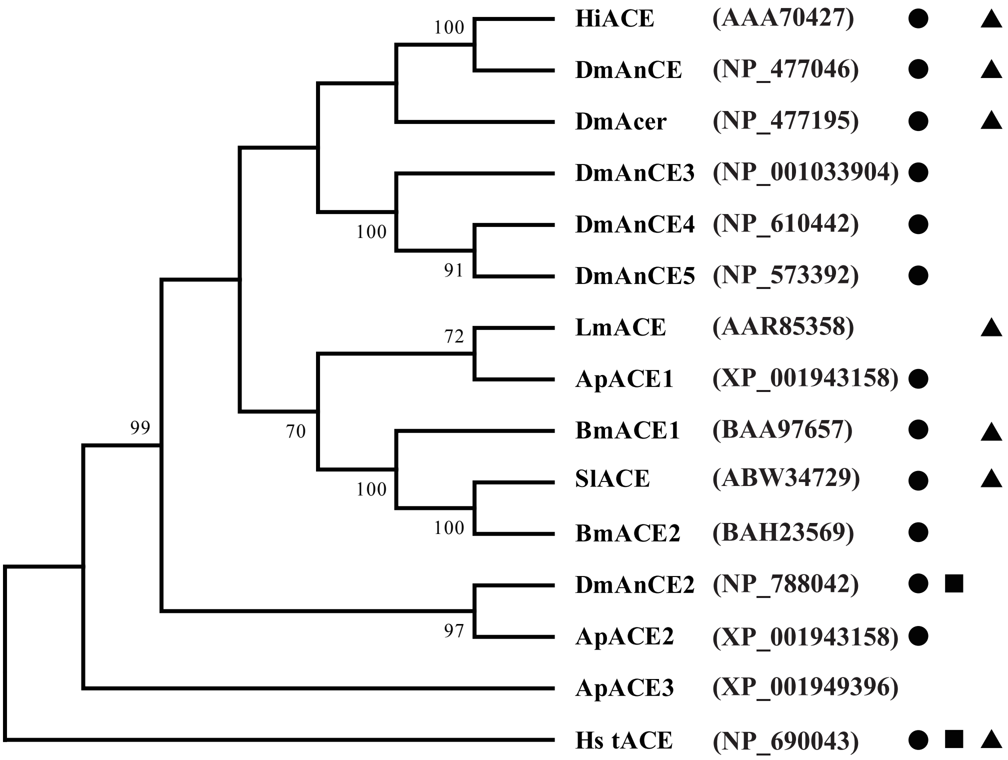

Angiotensin-converting enzymes modulate aphid – plant interactions

Wei Wang, Lan Luo, Hong Lu, Shaoliang Chen, Le Kang, Feng Cui

Figure S3

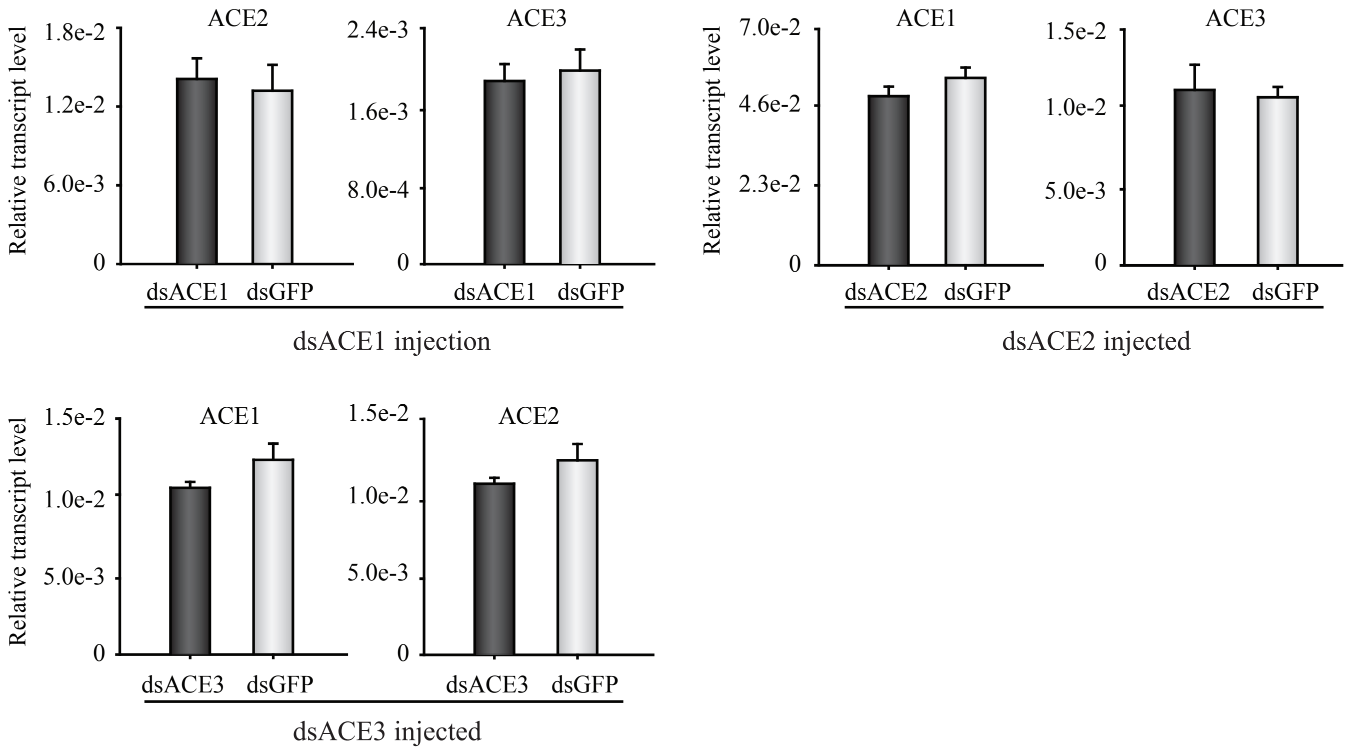

Angiotensin-converting enzymes modulate aphid – plant interactions

Wei Wang, Lan Luo, Hong Lu, Shaoliang Chen, Le Kang, Feng Cui

Figure S4

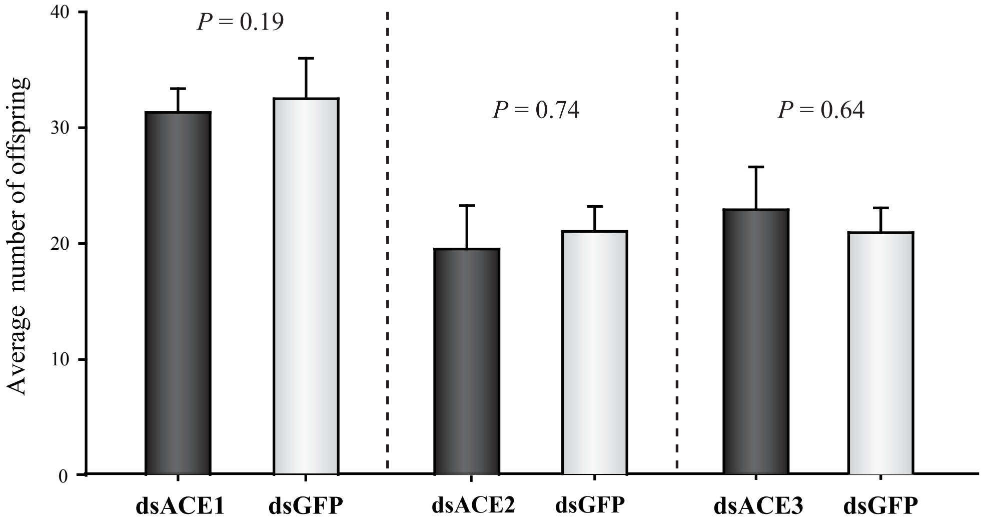

Angiotensin-converting enzymes modulate aphid–plant interactions

Wei Wang,[1,2#] Lan Luo,[1#] Hong Lu,[1] Shaoliang Chen,[2] Le Kang,[1] Feng Cui[1*]

Table S1. Primers used in the study

Primer name	Primer sequence	Product length (bp)
ACE1-F	ATGACGATGAAATTATATCATTTGTG	1914
ACE1-R	TTAAGGTTGACTTGTTTCACCCT	
ACE2-F	ATGAACGCCTTGACGACGA	1884
ACE2-R	TTACCCAATGTAAGGAATGTTGG	
ACE3-F	ATGAAGACTGTTCTAGTATTTGCA	1785
ACE3-R	TCTCAATATTCGACCAGCTTC	
ACE1-qPCR-F	TAAACAATTACACGGATA	214
ACE1-qPCR-R	TGCGCCCACATATCACCGAG	
ACE2-qPCR-F	GCACTACCACAAGAACAAC	286
ACE2-qPCR-R	CAGACGCATCCGATACAT	
ACE3-qPCR-F	TGTAATCGCTGATACTCCTT	217
ACE3-qPCR-R	AATATGTCCTGCTGCTGTA	
L27-qPCR-F	TCGTTACCCTCGGAAAGTC	108
L27-qPCR-R	GTTGGCATAAGGTGGTTGT	
ACE1-dsRNA-F	CGTACCTGCGACGTTGACG	313
ACE1-dsRNA-R	GGTTTGTCCACTGGGTCAATG	
ACE2-dsRNA-F	GCCAATTCAGTCTTAGAGGA	468
ACE2-dsRNA-R	GCCAAGTCGTTCTCGTAA	
ACE3-dsRNA-F	GCAATAGAGTGGCGATGG	330
ACE3-dsRNA-R	CTGGTTCAAGACTTAGTTCAC	
GFP-dsRNA-F	CACAAGTTCAGCGTGTCCG	420
GFP-dsRNA-R	GTTCACCTTGATGCCGTTC	
